# Identification of food insecurity factors in French-speaking Belgium: a qualitative study

**DOI:** 10.1186/s12889-019-7860-4

**Published:** 2019-12-05

**Authors:** Vincent Huberland, Pascal Semaille, Nadine Kacenelenbogen

**Affiliations:** General Practitioner, Department of General Medicine, Université Libre de Bruxelles, Brussels, Belgium

**Keywords:** Food insecurity, Food, Diet, Social determinants of health, Belgium, Poverty

## Abstract

**Background:**

Diet is an essential determinant of health. Among the health determinants, we find access problems that are summarized as food insecurity. While such food insecurity has been studied in other countries and correlated to several health problems, it has been scarcely assessed in Belgium. The purpose of this work was to determine the factors of food insecurity existing within the Belgian population.

**Method:**

From November 2016 to February 2017, a qualitative study using semi-structured interviews with 19 adults present attending the waiting rooms of six Public Social Action Centers in French-speaking Belgium, analyzed by grounded theory.

**Results:**

In Belgium, for given food preferences and needs, food insecurity could be summarized as inadequacy between necessary and available resources within two dimensions: at the access level, for financial, temporal, informational, and freedom of action, and at the food use level, for temporal factors, material, knowledge, and skills. In these situations of inadequacy, participants reported finding either strategies to restore balance, or being forced to alter the quality or quantity of their diet.

**Conclusion:**

While several factors of food insecurity may exist in Belgium, it appears essential that the first line of care these factors into consideration, since they could interfere with care and health, and because the first line of care is ideally placed to inform and refer the patients in question. Several courses of action are proposed in this work, which must still be confirmed by other studies.

## Background

According to the latest edition of the ‘Global Burden of Disease Study’, in 2016 [1], 18.8% of deaths worldwide and 9.6% of disability-adjusted life years (DALY) lost each year were attributable to inadequate food. In Belgium, these numbers were 14.7% and 8.5, respectively [1]. Therefore, food is one of the main determinants of health.

The diet of an individual is determined, in particular, by access to healthy food. Among other things, this is referred to as “food security”, defined by the United Nations for the Food and Agriculture Organization (FAO) as a “situation that exists when all people, at all times, have physical, social and economic access to sufficient, safe, and nutritious food that meets their dietary needs and food preferences for an active and healthy life” [3]. The absence of food security defines “food insecurity” (FI). This includes food insufficiency as well as all situations wherein access to food is not guaranteed and wherein such insufficiency is averted by various behaviors, often with adverse health effects [2]. In practice, the definition is extended to all situations wherein access is not perceived as guaranteed, whether this perception is consistent with the objective facts or not, since it remains likely to lead to the same compensatory behaviors [4]. The concepts of food security and FI can be applied using various system scales (country, household, individual, etc.). In this work, we will focus on the points of view of the individual and the household, which directly concern clinicians.

Several tools for identifying and measuring FI have been developed so far. In a systematic review of the literature by Ashby et al. [4, 5], the authors compared these tools and reported that the Household Food Security Survey Module (HFSSM, Additional file 1), used by the United States Department of Agriculture (USDA) in its annual surveys, is currently the most relevant tool available for FI diagnosis and measurement, primarily due to its recognized validity in psychometric studies, reliability, and correlation with weekly food expenditure, income, and distance from the poverty line. Moreover, it is the most recognized tool throughout the world and the most widely used at present.

Based on these tools, FI has been studied and followed up for many years in several countries [4]. With regard to Europe in general and Belgium in particular, the problem, on the whole, has been little studied [6]. In 2015, the first study involving an entire European population using the HFSSM was carried out in France; this work estimated the prevalence of FI due to lack of access to food for financial reasons to occur for at least 12.2% of the population [7]. For Belgium, the best estimate currently available is Eurostat’s food poverty indicator: in 2016, 6.1% of households in Belgium declared that they could not afford to buy every other day a meal including meat, chicken, fish, or a vegetarian equivalent [8]. Food aid figures also provide an estimate of the magnitude of the problem. In 2015, 138,557 people used at least once the services of the Belgian Federation of Food Banks. This number has been growing steadily since 2005 [9].

These estimates display many limitations, such as that the indicators used only take into account some of the FI dimensions. Food security is typically identified by four dimensions, each depending on the previous one: sufficient production or availability, sufficient access to food, adequate utilization, and stability over time. A lack in any of these dimensions presents a risk of FI [5].

For each of these dimensions, several factors have been highlighted in the international literature so far. A number of these factors are quantitatively well-established, such as the financial factor. Several articles have reported that the higher price of healthy food forces people with financial insecurity to turn to a lower-quality diet [10, 11]. Among the factors defining food security that also correlate with diet in several studies are: lack of quality food within a reasonable proximity [12, 13], nutritional and culinary skills [14], and coercion, as in the case of financial coercion in households with intimate partner violence [15, 16].

In exclusively qualitative studies, other factors have been proposed. These include pace of life and lack of time [13, 17], personal mobility, information level, social relationships, lack of motivation, precarious housing, and lack of kitchen equipment [17].

Currently, there is no qualitative or quantitative scientific synthesis for Belgium in regard to these factors. The only available summary is included in the “White Paper for Universal Access to Quality Food” [17], published in 2015 by the Socialist Mutuality and based on a few focus groups and the editors’ expertise, primarily interested, first and foremost, in finding solutions.

To date, there is not one single overall indicator that includes all of the previously identified FI factors. The currently existing tools mostly ask very general questions or only take into account the financial dimension of access to food, as in the HFSSM. Only the Radimer/Cornell questionnaire accounts for physical capacity to shop or cook food, without considering other items. All these tools, therefore, probably underestimate the FI prevalence [4, 5]. In their literature review [5], Ashby et al. insisted on the need to take other dimensions into consideration in questionnaires, including availability and other aspects of access (especially geographical). The authors stressed that the current tools have mostly been validated in vulnerable populations and, as such, may be less valid in the general population.

The lack of quality data reflecting the complexity of the situation is in contrast with the many correlations that are currently highlighted between FI and certain health problems likely to be consequences of FI.

In adults, FI has been correlated with, among other things, the risk of diabetes [18], cardiovascular risk [19], intimate partner violence [20], and, overall, diminished perceived health [21]. In children and adolescents, FI has been correlated with the risk of psychomotor developmental delays, certain behavioral disorders, anxiety and depressive disorders, and suicidal ideation, as well as academic performance [21, 22].

For those possible consequences, a proposed causal mechanism is that in the concerned individuals, FI ​​might contribute to significant mental stress, inducing a less diversified diet centered on foods rich in energy, poor in other nutrients, and cheaper per calorie [2]. It is paramount to note that despite the correlations described, the causal links between FI and those problems remain complex and mostly hypothetical in most cases. For example, intimate partner violence has been proposed as both a cause and consequence of FI: in a household with intimate partner violence, financial coercion could render it more difficult for the abused partner to buy food; however, the stress of living in a food insecure household could also increase aggressivity [15, 16, 20].

Several studies have also specifically focused on the implications of FI for the first line of care, i.e. for screening and management [23] as well as its interference with the management of other health problems and chronic diseases like diabetes [2, 18].

### Objectives

FI is likely to be a significant health problem that should be considered in our practices. For Belgium, given the lack of quality information on this subject, our first aim was to determine the extent of the problem as well as its implications within the Belgian population. Therefore, the main objective of our study was to identify the FI factors present in the Belgian population and how they interact with food security.

In order to later quantify FI and the identified factors, the secondary objective was to create a more comprehensive and population-specific tool that could then allow us to quantify food security in the Belgian population.

## Methods

### Interview matrix and questionnaire

In order to identify all the FI ​​factors prevailing in the Belgian population, we have conducted a qualitative study, using individual semi-structured interviews. Semi-structured interviewing renders it possible to explore all the dimensions and interactions of a complex topic while also opening space for new information. Therefore, this was an interesting method for our aim to identify and describe all FI factors in the population. Since the subject of the study is related to poverty and, as such, may be emotionally charged and associated with feelings of shame and stigmatization, we chose to work in individual interviews rather than groups. For these interviews, an interview matrix was created based on the literature review.

As our research also aimed to propose the basis for an FI identification tool in Belgium, our interviews included the administration of a questionnaire (Additional file 1). This questionnaire was established on the basis of the HFSSM questions [4, 5] including financial factors and questions from other questionnaires [5], along with the FI factors identified in the literature for the other topics, such as a general FI screening question, food availability, physical and geographical access, equipment, time and ability to use, knowledge, aids, and future perspectives [12–17].

### Sampling

This study was centered on the area covered by our department’s interventions, which is French-speaking Belgium.

In order to identify a maximum number of FI factors present in the French-speaking Belgian population, we tried to select a sample comprising a maximum of people in an FI situation. By extrapolating the Eurostat statistics [8] and studies carried out in other countries [7], we obtained an estimated FI prevalence of 5–10% in Belgium. This prevalence a priori made it physically impossible to directly sample the entire population, so we identified a sub-population in which we thought we could find all the FI factors present in French-speaking Belgium, yet with a higher prevalence. We thus chose the population of users attending the waiting rooms of the Public Social Services Centers (CPAS), within which we found all the FI problems existing in the general population, though with a higher prevalence. Of note, the health problems encountered in situations of poverty are generally similar to those of the remaining population, but more prevalent [24]. This population offered the advantage of being clearly defined and easily accessible.

For the sake of completeness, we sought to create as diverse a sample as possible. We conducted our interviews in the CPAS of several Belgian municipalities, assuming that the geographical factor could be influential, among other things, in regard to the potentially determining nature of distances, mechanisms of solidarity and charity, and public policies [17]. Thus, we conducted our study in several municipalities of the capital (CPAS of Etterbeek, Marolles, Neder-Over-Heembeek), in other cities (Namur and Charleroi), and in a rural area (Neufchâteau). Rurality was defined according to the OECD and Eurostat criteria [25].

Participants were then selected through maximum variation sampling using the following potentially influential diversification criteria: age, gender, household composition, occupation, income level, presence of a particular diet, and residence status in Belgium.

In the CPAS concerned, people attending the waiting rooms were invited individually and randomly to participate in our study. For those subjects who accepted the invitation, the interview took place in the morning in a room made available by the CPAS, with only the participant and interviewer present. The inclusion of new participants continued until the diversification criteria were fully met and there was external saturation of the data.

### Data collection and adaptation of the matrix and questionnaire

The interviews were conducted face-to-face, based on the established matrix, using a semi-directive methodology, with the examiner only intervening to re-frame the discussion, delve deeper into certain issues, and tackle new topics. Following each semi-structured interview, our questionnaire was orally administered to the participant. Each entire session was recorded by dictaphone, and the interviews were then transcribed.

The interview matrix and the questionnaire were revised throughout the interviews based on the understanding of the questions, the relevance of the answers obtained, and the appearance of new topics.

The final questionnaire version was elaborated by adding the different items of the theoretical framework from the interviews. For the formulation of the questions, we left the sentences stated in the interviews for the item concerned in order to get closer to the participants’ experience.

### Data analysis

The analysis of the various interviews’ transcripts was carried out via the anchored theory [26], solely on the basis of the interview transcripts by the principal author alone. The content of the various transcribed interviews was first coded by text unity according to the topics discussed and the ideas expressed upon these topics. The different links between the ideas expressed were then coded. This work was performed using Atlas.ti software, Version 7, except for the identification of connections between the different topics, for which the work was carried out manually.

The results of the qualitative analysis were interpreted using the questionnaire responses. The data from all the questionnaires were aggregated as solely descriptive statistics using the LibreOffice Calc spreadsheet software. The interpretation was then discussed among the authors alone.

## Results

In total, 18 interviews were carried out during 13 mornings in six CPAS from November 9, 2016, to February 24, 2017. We met a total of 19 study participants, two of whom participated together in the interview. The interview duration was 13–45 min, with a median of 27 min.

### Participants’ profile

The participants’ profiles, based on the study’s diversification criteria and other relevant socio-demographic variables, are shown in Table 1. Among the 19 participants, there were people in all age groups from 18 to 65 years, 10 men, and nine women. There were four single people, eight couples with children, three single mothers, a single father, and three people living with other people who were not direct relatives. Overall, nine people were in a relationship, and 11 had dependent children. There were three unemployed people, two students, one retired person, and one handicapped person; the others had no special status. None of the participants had a paying job. Total monthly incomes ranged between all levels considered, with a median of € 1000–1500 per household. Among the participants, 14 did not report having a particular diet, one was vegetarian, two consumed halal food, and one followed a diet for diabetics. A total of 10 participants were Belgian, three were Europeans from outside Belgium, and six were non-European nationals. Regarding residency status, only one participant was in an illegal residence situation.

**Table 1:** Participants’ profile

		Number (Total = 19)
Place	Capital city	7
	Other city	8
	Rural area	4
Age (years old)	18–25	3
	26–35	4
	36–45	7
	46–55	1
	56–65	4
Gender	Male	10
	Female	9
Household	Single adult	4
	Couple with children	8
	Single mother	3
	Single father	1
	Cohabitants	3
Origin	Belgian	9
	Europe except Belgium	3
	Africa	6
	Asia	1
Stay in the neigborhood	< 1 year	4
	1–10 years	5
	Since birth	1
	Missing	9
Legal stay?	Yes	18
	No	1
Household monthly income in Euros	< 500	3
	500–1000	5
	1000–1500	6
	1500–2000	4
	> 2000	1
Highest degree	Before secondary school	4
	Secondary school	11
	Higher education	3
	Missing	1
Status	Unemployed	3
	Student	2
	Retired	1
	Disabled	1
	None	12
Housing	Owner	2
	Renting	11
	Homeless	2
	Cohabitant	1
	Other	2
	Missing	1
Special diet	Vegetarian	1
	Halal	2
	For health reasons	1
	None	14
	Missing	1
Somatic disease	Yes	12
	No	6
	Missing	1
Mental health problems	Yes	4
	No	14
	Missing	1
Perceived health status	Bad	2
	Average	7
	Good	5
	Very good	3
	Missing	2

Although the possibility of using translators for the interviews was provided, all those who agreed to participate had the necessary skills in French to take part in the interview in this language. All the people to whom we spoke who were not fluent in French declined to participate.

Four participants had not completed their secondary studies, 11 had only an intermediate-level education, and three had higher degrees. Overall, two participants owned their residence, 11 were renters, one had a roommate, and two were homeless.

Among the participants, 12 described somatic health problems (high blood pressure, obesity, diabetes, hypercholesterolemia, ischemic cardiomyopathy, atrial fibrillation, aneurysm rupture, herniated disc, tendinitis, asthma, chronic obstructive pulmonary disease, peptic ulcer, or anemia), and four reported mental health issues (depression or anxiety). Two people rated their health as poor, seven as average, five as good, and three as very good.

#### Food situation

Among the participants, 10 lived in an FI household according to the HFSSM, including three with a very low food security level. Based on the interviews and questionnaires, 16 of the 19 participants stated they experienced at least one obstacle to access of a healthy diet.

### Semi-structured interviews

Overall, 16 out of the 18 interviews reached internal saturation, while the remaining two had to be shortened in order to allow the participants to leave for another appointment. As only one new topic was highlighted in the last 10 interviews, we determined to have reached external saturation.

**Fig. 1:**
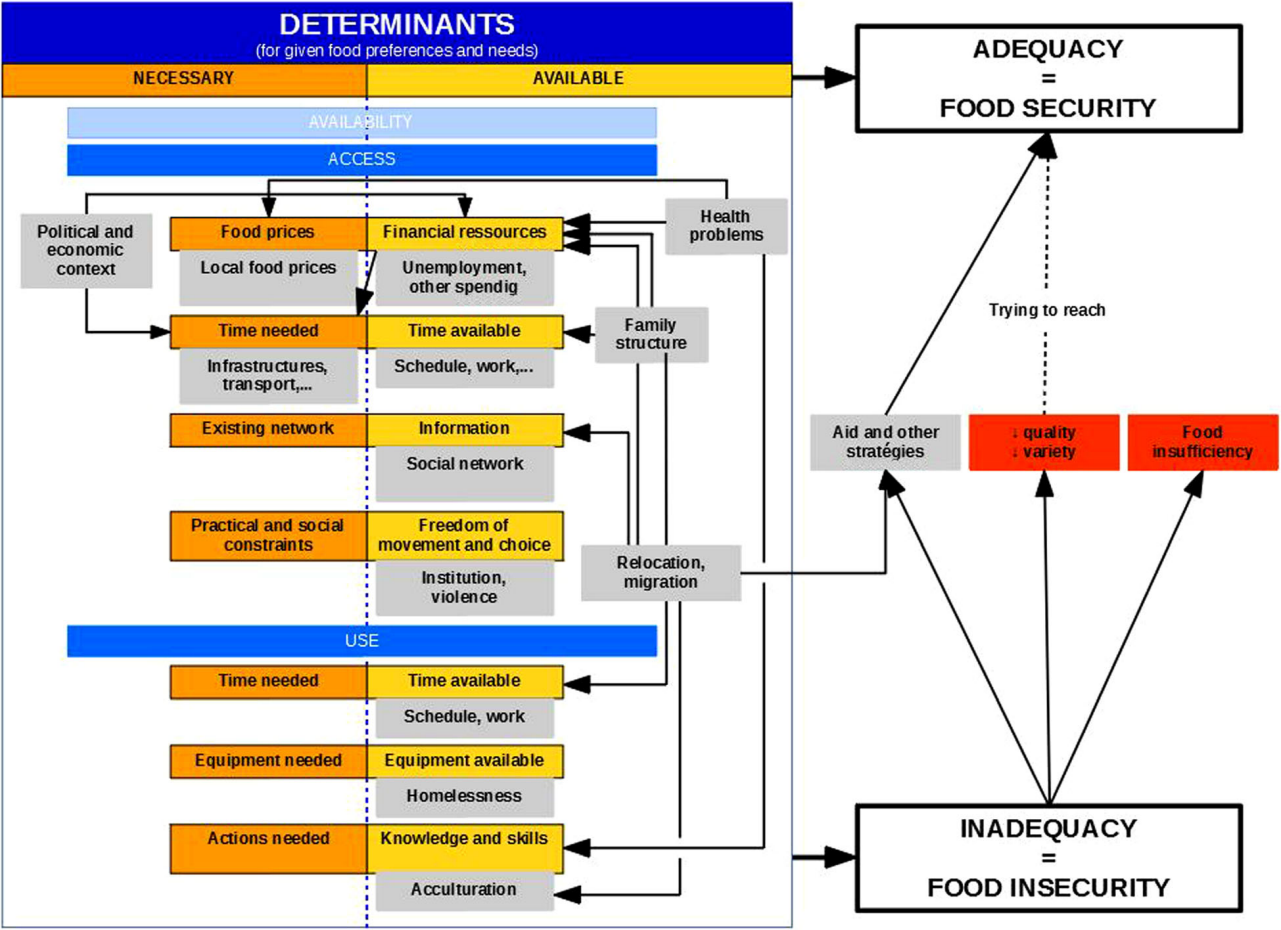
Theoretical framework: Food insecurity (FI) factors in Belgium. Legend: FI factors prevalent in Belgium with their underlying mechanisms, according to our study results. The expected, though absent elements are transparent. Grey squares represent mechanisms interacting with these FI factors

The participants described numerous mechanisms limiting access to healthy eating. These were synthesized in a theoretical framework, inspired by, among other things, the theoretical framework proposed in the “White Paper” of Socialist Mutuality [17] and in the definition of the Rome Declaration [3], as reproduced in Fig. 1. For each participant and in general, the mechanisms involved could be summarized as inadequacies between the means available to the individual and those necessary to acquire a diet corresponding to their preferences and needs. These inadequacies ultimately involved seven factors, which were grouped into two of the four dimensions conventionally recognized for FI: access to food and utilization. No participant expressed difficulties directly relating to food availability. In addition, since the interviews considered a period covering at least the last 12 months, the dimension of stability over time was judged to be included from the outset in the other dimensions. In terms of access to food, we found the adequacy between food prices and financial means, time needed and time available, the existing network and its knowledge, practical and social constraints to access of food, and the freedom of choice and movement the individual held. Finally, at the utilization level, there was adequacy between the time needed and the time available, the material needed and available for cooking and preservation, and the skills and abilities needed and available for food preparation. Inadequacies present in one of the cited dimensions limited access to adequate food and, therefore, defined FI.

In this setting, several strategies concerning attempts to return to a food security situation were described. These included the use of informal or institutional aids, organizational changes, and changes in diet. In several cases, the FI ​​situation required a less healthy diet, such as reduced food quality and portion size or skipping meals.

#### Food insecurity factors

##### Preferences and diets

Ten of the interviews highlighted the role of preferences in food security (Table 2*)*. These were mainly described as determined by various cultural factors, such as family education, geographical origin, and impact of advertising and social pressure. These preferences could be ill-defined, such as a country-specific diet or focus on organic farming as a definition of quality food. They were, at times, more precise, as in the case of strict diets defined by convictions (vegetarian, halal) or health reasons (diabetes diet). Two parents described their children’s preferences as playing a very significant role in household food choices, as their kids had more limited tastes and were more vulnerable to external influences like advertising (Table 2).

**Table 2:** Preferences and diets

Table 2.1	
“So, well, if there are Dannon products on sale, let’s say, he does not want it because the packaging is not as attractive as the other, because it doesn’t have the little spoon or the little tattoo that the other one has.” (Interview n°13)	
Table 2.2	
“I find that vegetarian food, especially, is too expensive for even one person. For example, if my whole family became vegetarian, we would not be able to eat anymore.” (Interview n°5)	
Table 2.3	
“- if my budget allows me to ... eat well, then I do not skip on the means, but if at the end of the month, there isn’t much left in my wallet, I’ll have to either restrict uh ... eat less, okay? (…) As I told you, I prefer to eat less, but always good quality.” (Interview n°8)	
Table 2.4	
“- Participant: No, in Syria, myself, I... When I was in Syria, I ate a lot more vegetables than I do in Belgium. In Belgium, vegetables... I think that vegetables in Belgium have no taste. (…) Investigator: (...) And, therefore, you tell me that you eat less vegetables here? What are you eating then, instead? Participant: I’m eating, uh... Normally, I eat potatoes, fries, at home, or some, how do you say... My wife cooks pizza, too, (...)” (Interview n°17)	

These food preferences were not described as limiting access to adequate food for them but as making them more vulnerable to multiple barriers. Several preferences were described as decreasing the possible choices and, therefore, resulting in constraints, such as higher expenses per meal or the need to purchase in several stores or specialty stores. This could, for example, increase the budget and time needed for purchases, rendering them more vulnerable to financial and time factors. Of note, these factors will be described in more detail hereafter. (Table 2) These preferences could be an essential determinant governing the choice between qualitative and quantitative reduction in the diet in limited access situations (Table 2).

According to two interviews, food preferences along with culinary knowledge and skills could play a key role in food security in acculturation processes. This issue concerned people who had acquired certain food preferences, knowledge, and skills in another cultural setting. When they reached their new setting, they stated that they had not found the possibility of resuming a healthy diet, that the products of their original setting were difficult to access, and that their preferences and culinary skills limited their ability to adopt a healthy diet with local products. They described having turned to products that were easily accessible, both materially and culturally (Table 2).

##### Access to food

The access to food dimension was mentioned in all the interviews. Many mechanisms were described as leading to FI situations due to inadequacies in financial, temporal, informational, or freedom factors.

##### Financial factors

Financial limitations regarding access to healthy food were highlighted in 17 interviews (Table 3). These could be summarized as an inadequacy between two determinants: the financial means available to the consumer and food prices.

**Table 3:** Financial factors

“**-** Participant: (...) with my unemployment, when I have paid my bills, I have less than 300 euros a month for food. (…) So, on a daily basis, my expenses in food, range from €5 to €7.50. If I need clothes or cleaning products, then there is less for food. (…) According to the purchases, if there is ... a cleaning product or if there are clothes to buy, well, it comes to a simple, a simple, little 365 brand 400-g lasagna to warm up in the microwave.” (Interview n°11)	
“- Fresh? We don’t know. Except at the beginning of the month, when we can, we do the shopping, we take a salad, a cucumber, tomatoes, potatoes, and all that, onions, but then, at the end of the month, it’s the glass jars, cans, you know, the preserves. (…) Midway, before, from the 15th, well, 15–16, we start on the jars and preserves, the junk food, the fast food places, and the stuff that does not cost much, you know.” (Interview n°10)	
“So, uh ... I have all the debts falling on me, so I have three kids to feed so, uh ... I’m going to feed ... uh ... There are evenings where it is just eggs, because eggs, well, they are cheaper, and they are still protein. So, eggs, bread, and that’s it! Sometimes it is pasta with butter, all white. But hey, they won’t go to sleep on an empty stomach anyway. Me, it happens to me, yes, but hey, it does not matter, I have reserves, but, well, them, no...” (Interview n°13)	

The 17 participants expressed difficulties in accessing healthy food because of insufficient financial means to meet their household’s food needs and preferences. This was attributed, on the one hand, to too low income (e.g.*,* due to unemployment or disability or to insufficient CPAS support) and, on the other hand, to too many other expenditures. For 12 people, food played a secondary role in the household budget after other expenses such as rent, bills, possible debts and maintenance payments, health care, addictions, and even pets. This situation was described as subject to cyclic variations during the month, with the diet quality decreasing as the monthly income was depleted, or during certain seasons, with heating bills increasing the expenditure constraints in the winter. For two households with children, the various expenses relating to children and their well-being were described as priorities, leading to food deprivation for parents.

Furthermore, 12 interviews also highlighted limitations from the supply point of view. These participants found food prices too high, especially for foods such as meat, fish, vegetables, and products of higher nutritional and culinary quality in general.

Finally, the interviews revealed that in a context of limited financial means, the price difference depending on the quality of the food renders it necessary to make certain choices. Thirteen people described having been forced to reduce the quality of their diet, either by depriving themselves of certain foods and, therefore, reducing the diversity of their diet or by buying products considered to be cheaper and of lower quality, such as generic and industrial products. In other cases, six participants explained that they had to reduce the amount of food purchased, either by reducing portions or by skipping meals (Table 2).

##### Household structure, living environment, and housing (Table 4)

Nine interviews highlighted their household structure as playing an essential role. Life in a group, as a couple, or with other people generating income was presented as a protective factor in many ways. It facilitates wholesale purchases, which are often cheaper per unit than the retail price, limiting expenditures and, thus, increasing variety (Table 4). It allows for the sharing of the goods, increasing the culinary possibilities and, consequently, the dietary diversity (Table 4). It also limits the time required for cooking or shopping (Table 4). Conversely, living alone or with other dependents was described as an FI factor, leading to greater financial and time expenditures (Table 4).

**Table 4:** Household structure, environment, life, and housing

Table 4.1	
“- Here, for one person, let’s say, we cannot buy something in bulk and say, well, we’re going to divide this over several days because, here, we just... So that, we will say, it causes more redundancy in what we will eat than if, here, we cooked for two or three.” (Interview n°15)	
Table 4.2	
“So ... uh ... there, well, like ... uh ... so, by settling [in the town], I ended up with a roommate, so, all that was needed was there, so, yes, to answer honestly, yes, um ... there is no problem.” (Interview n°15)	
Table 4.3	
“- Participant: Ah. No money, no work, no money. - Investigator: But then, to buy food, how does that go? - Participant: Uh... With my brother, from time to time, he goes in...” (Interview n°12)	
Table 4.4	
“- I come back once after school, so uh ... well, there is something ... well, an hour, to quickly prepare and everything for it to be okay. So, things are going to be pretty, uh, quick to prepare. (Interview n°13)	

Not having a home was a major source of FI because of the lack of material necessary for the preparation and preservation of food as well as the impossibility of being admitted to certain institutions and, therefore, not having access to certain aid.

##### Health status (Table 5)

The presence of mental or somatic health problems was regularly identified as a limitation to access to a healthy diet in 11 interviews.

**Table 5:** Health status

« - (…) So, when I go shopping, with my cart, because if I have to carry a bag, it’s even worse for my leg, it takes me an afternoon, uh... two or three times a week.” (Interview n°110)	
“- Investigator: Yes. And you said to me, sometimes, you are much too tired by the end of the day. - Participant: It’s especially irritating, stressful. (…) No, at that moment, I’m doing something super fast and, they eat, they go to sleep, they do not care. (Interview n°13)	

Health problems were described as having an impact on the food budget. This could be indirect, through the cost of care and treatment, but also direct, with symptoms like fatigue and anxiety leading to the consumption of certain products or foods (such as fast foods, prepared meals, and sweets), which represent additional costs in the household budget. Some people also described a vicious circle in which the use of certain substances (cannabis, heroin) decreased the feeling of hunger.

Health problems were likely to reduce the time available for shopping and food preparation. This could be attributed to the time required for care but also to a decrease in functional abilities increasing the time required for shopping and food preparation.

##### Geographic factors

None of the participants expressed an inability to find healthy food within a reasonable distance from home (Table 6). However, three participants explained that the food currently available in their neighborhood was too expensive, so they were forced to travel, sometimes dozens of miles, to find an affordable diet. These geographical limitations were described as increasing the time and expense required for a quality diet. In some areas, lack of transportation seemed to limit the ability of transporting large quantities, limiting choices and increasing spending, with wholesale purchases often less expensive per unit.

**Table 6:** Geographical factors

“- Participant: Sometimes I was in Auchan (…) to buy the...fruit and the vegetables. It is cheaper. (Interview n°2)	
“Sometimes, I’d like to buy this or that, but I know I cannot carry it.” (Interview n°13)	

##### Political and economic framework

The role played by the political and economic framework in personal situations was mentioned in three interviews (Table 7). The participants explained that the concentration of resources by a minority of individuals has led to an increase in inequalities, which could possibly be solved through political measures.

**Table 7:** General political and economic framework

“- It is, in any case, it is the rich who... It is always the rich who have more and the regular people who have less.” (Interview n°13)	

##### Time constraints

Overall, 16 interviews highlighted the impact of time constraints on diet (Table 8). These constraints concerned the dimensions of access to food and utilization. They could be described as limiting in situations of inadequacy between the time necessary and time available for purchasing and preparing the food. The time needed could be described as increased by constraints related to a particular diet or health problem (Table 6). Concerning financial resources, the time spent on food appeared to be positioned after other priorities and constraints in scheduling. These priorities could include heavy or shift work schedules, major trips, appointments, administrative procedures, or family obligations. These time constraints could be perceived as forcing subjects to be satisfied with meals that are easy to cook or to skip meals.

**Table 8:** Time constraints

“- [Speaking of Restos du Coeur (Charity that offers free meals)] Unfortunately, there are days that we don’t manage to go, because, well, I have appointments in the hospital and everything, often at noon time and so ... we do not always manage to go, you know.” (Interview n°1)	
“- Well, we don’t get many varied meals, I think, because I always try to do it faster, I make things that are really easy to make: pasta sauce, etc. - really easy things.” (Interview n°5)	
“- (...) So, I started on Sunday evening, I left at 10–11 p.m., midnight, I drove, I unloaded, (...) That was also a problem, for example, wanting to eat something hearty at three in the morning, you know. I knew guys who ate steaks at three in the morning or a chicken, huh.” (Interview n°9)	

##### Information

In six interviews, lack of information about the food supply in the neighborhood or region where the individual lived could be perceived as a FI factor (Table 9). Six participants described a change in setting*,* e.g.*,* during a move, migration, or release from prison, that resulted in limited access to food, both directly and indirectly. Directly, not knowing the neighborhood or region when they arrived, the participants described having only a limited number of possibilities that provided food. They had limited food choices and often turned to easy solutions (prepared meals, supermarkets, or fast food). Indirectly, in six interviews, the lack of knowledge of the network and impoverishment of the social network brought about by the change of framework also limited the possibilities to find work or housing, to find support networks, or to access certain aid. The negative consequences of a change in setting could be limited by strong links with people who understand the new framework and pass on their knowledge.

**Table 9:** Information

“- [Speaking of the food situation, returning from Rwanda] Uh... I think it’s going to get better from the moment I get into new habits, well, just, um, going shopping, and knowing, well, where do I get x or y products, I think it will improve (...)” » (Interview n°15)	
“- This is a nightmare. And besides, me, I ... since I’m [in this municipality], I’m not at home. (…) And then, I don’t have much luck: I had an accident when I arrived here, at the lake, because I broke my back and then, I had an accident with my car, so I really could not, um … get myself out of it.” (Interview n°10)	
“- Investigator: But when you arrived in Belgium, you have, how have you gotten food? - Participant: My husband (...), he’s been in Belgium for 38 years, yes.” (Interview n°2)	

##### Practical and social constraints and freedom of action (Table 10)

Not having freedom of movement and choices was described as a FI factor in two interviews. The factor of being dependent on a closed environment or institution (school, prison, etc.) for food was described as a source of constraints limiting the choice of food and, thus, the choice of a healthier diet. In addition, one of the participants mentioned the notion of violence as a food limitation, citing a case wherein food was stolen by a person in an authority position in the food distribution chain.

**Table 10:** Practical and social constraints and freedom of action

“- The concern I have is that the children, in fact, uh ... are in school at noon, so they do not eat healthy, I don’t know.” (Interview n°8)	
“- Participant: Of course, they bring us what we have, what we in prisoners’ jargon, we call “chow”, but, fortunately we also manage to cook our own stuff in the cell. (...)Well, it’s not bad or good, the thing is, well, the supervisors, on the way, they have their own meals.” (Interview n°7)	

##### Utilization

Limitations in utilization were mentioned in six interviews, possibly leading to FI situations due to a lack of materials or of the knowledge and skills necessary for proper food utilization.

##### Kitchen and storage equipment

Not having the necessary equipment to store and cook food, e.g., due to being homeless, was described as limiting the possible food choices in six interviews (Table 11). The factor of not having preservation equipment (refrigerator, freezer) greatly limited the possibility of buying fresh products. The factor of not having utensils forced the purchase of prepared meals or fast food, all likely to increase food expenses, given that fast food is relatively expensive.

**Table 11:** Kitchen and storage equipment

“- Investigator: And to preserve the food? - Participant: It’s in boxes, outside, as long as it freezes. When it does not freeze, well, then... it’s more complicated.” (Interview n°1)	
“- when we are on the street, we do not know how to eat, we do not know how to cook, so we have to buy crappy take-out, and junk... And that, it costs a lot for what we eat, for what we have in the stomach!” (Interview n°7)	

##### Knowledge and skills

Food knowledge and skills were also reported as important factors in two interviews (Table 12). Theoretical knowledge of what a quality diet actually represents was highlighted as a necessary means to access a healthy diet. Being able to prepare several types of dishes was highlighted as a way to diversify the diet and, therefore, have a quality diet.

**Table 12:** Knowledge and skills

“Well, healthy, it’s a good piece of meat, vegetables, uh ... good cooking with butter, that’s it. For me, this is not healthy in the sense of “diet”, but for me, it’s healthy because it’s good and it is, it’s... Well, with butter, with a good piece of meat and vegetables, you have everything you need in your body and ... and there you are, you sleep well, you are not hungry and... that’s it.” (Interview n°6)	
“- Participant: But otherwise, yes, uh, either, if I do it quickly, well, I make spaghetti bolognese, uh ... otherwise, I make meat, with pasta or potatoes with vegetables, you know. Finished, that’s it.” - Investigator: And is that at every meal? - Participant: Yeah, because, me, I’m not a great cook.” (Interview n°14)	

##### Strategies adopted by the participants Spontaneous strategies (Table 13)

Eight participants described strategies to limit the impact of the various factors mentioned, especially the financial factors. These included strategic budget management, low-cost purchases *(*e.g.*,* in-store, seasonal products, cheaper stores, or multiple stores), purchasing in bulk, cutting back on other expenses (including recreation and various pleasures), setting aside reserves in periods of relative abundance, optimizing the utilization of available food, and production of one’s own food (e.g.*,* with a vegetable garden). Three participants described being forced into other illegal or unethical strategies, such as begging, theft, non-payment of bills, or performing undeclared work.

**Table 13:** Spontaneous strategies

“- The fact that we buy for two and divide the shopping into two. Well, for one, it’s cheaper, huh, when you buy it. For example, we have two plus a free one, good, well, that’s a question about purchasing, organization too.” (Interview n°9)	
“-Well, I prefer to pay all my bills and really stock the house with food, so after that, we can do what we want with the money.” (Interview n°3)	
“So, thus, it happened that I, yes, I stole; now, I ... I avoid, but, well, when I have no choice, I do it. That’s, that’s survival for me, so, therefore, it’s not like someone who steals for pleasure. I steal in order to eat, huh. (Interview n°6)	
“- I go crazy, I say to myself: ‘How am I going to feed my children tonight? I have nothing, I have nothing. They will go to school without any sandwiches tomorrow.’ So what do we do? We beg. (Interview n°10)	

##### Network and aid

A total of 13 participants spoke of the protective role of informal and institutional or associative aids in FI (Table 14).

**Table 14:** Network and aid

Table 14.1	
“(...) I still have friends who still have money, good, who help me a lot, and if they go to the restaurant, and I have no money, they still take me with them and, good, they don’t ask me for anything afterwards. (Interview n°6)	
“- [Speaking of the family] So, there, for some time, I have not asked them anymore uh ... I see them a little less, because I try to let them be, I contacted today, you know, this organization [CPAS] for uh ... to solicit their help.” (Interview n°8)	
Table 14.2	
“- Investigator: (...) Did you ever, in the past, benefit from a food aid program, food parcels or anything else? - Participant: No, never, not me. Because I have a lot of pride, so, therefore, I never uh...” (Interview n°6)	
“- [Speaking of food aid] Because we must be, how ... a household composition ... and as we are removed from the municipality, we cannot get it...” (Interview n°1)	
“- Restaurants du Coeur, they salt, they pepper while cooking, so, it gives the impression at times that it is hospital kitchens, that’s it. So, well, me at the Restaurants du Coeur, I’m not going there.” (Interview n°7)	

In 11 interviews, having strong connections with family, friends, neighbors, or other acquaintances provided support in terms of food, money, or travel in case of limitations. The participants, however, emphasized the exhaustible and time-limited nature of such support (Table 14).

Institutional and associative aids were mentioned in 10 interviews. These included aid provided by the CPAS and various food aid systems (food parcels, restaurants, and social groceries). The CPAS was mainly mentioned for its administrative and financial aid, which was described as allowing the participants to maintain an income and, therefore, a minimum of access to food, though some participants expressed insufficient income and excessive administrative barriers. Out of 19 participants, five reported having received food aid within the last 12 months. This was described as increasing the frequency and variety of meals while also limiting food expenses, thus increasing the budget available for other expenses. Several FI people described not receiving food aid because of shame, ignorance, the presence of administrative barriers, or lack of appeal for these aids (Table 14).

#### Emotional and relational dimensions

On two occasions, the interview’s content appeared to provoke a strong emotional reaction in the participants (Table 15). This was always related to the relevance of preserving children’s food security.

**Table 15:** Emotional and relational dimensions

“- Well, it happened to me that I had only five euros on me, but, ha!... The children, that, they have to eat, it’s my kids, I’m responsible for them, so, I cannot afford to. It’s sad to say that, uh, in 2016 (with tears in her eyes).” (Interview n°13)	
“- Investigator: Okay. And at the level, for example, of the price of food, this plays an important role for you? - Participant 2: Yes. - Participant 1: Yes. Now, the food is too expensive, huh. (…) - Investigator: So, does that change the diet? - Participant 2: No. - Participant 1: No. » HFSSM: 7 and 6 non-negative responses, FI (Interview n°18)	

In three interviews, we noted an avoidance of the questions or a denial of the food situation: in those interviews, a participant who had denied experiencing financial limitations to the access of food in the semi-directed part of the interview was in FI status in their responses to the HFSSM.

### Questionnaire

During the interviews, new topics emerged, and several questions revealed to be too vague, ambiguous, or too far from the actual experiences of the study population were reformulated or deleted. In the final version, we kept all the items of the HFSSM and all the sections we had identified in the initial version but changed the phrasing of most questions for the questions to be closer to the words employed in the interviews. Regarding the response options, the multiple choice question “Was it often, sometimes, or never true for you in the last 12 months?” was changed to “In the past 12 months, has this happened: about once a year, about once a month, about once a week, or almost every day?” This formulation sought to enable a better quantification and gradation of the responses in order to possibly set thresholds thereafter.

In the final version, we added a new section based on the new factors identified due to the interviews. The section included questions about food prices, time, information, practical and social constraints, and skills.

The final questionnaire version is presented in Additional file 2.

## Discussion

In this study, we initially carried out an overview of the FI factors prevailing in Belgium. With the exception of age, our sample met all our diversification criteria. The study sample covered a wide range of ages, genders, health statuses, household compositions, housing, incomes, education, food preferences, nationalities, and living environments. Our sample did not include anyone over 65 years of age nor anyone unable to speak French.

The absence of people over 65 years old in the sample could be explained by their low presence in the waiting rooms of the CPAS, perhaps because they prefer to be seen at home. This absence may have deprived our results of some age-related FI mechanisms described in the literature, such as limitations in terms of food utilization and access and lack of physical or intellectual ability to shop for and prepare food [5]. Such health-related limitations in people under 65 years old were present in our interviews, but there might have been other age-related factors that we did not take into account.

Our sample did not include any participants unable to speak French because everyone with this profile refused to participate. In spite of ten people of foreign origin under various conditions of residence being present in our sample, this lack may have deprived our results of certain factors specific to migration contexts and cultural barriers.

Of the 19 participants, 16 reported experiencing at least one FI factor. For the most part, we had internally saturated interviews and, on the whole, achieved external data saturation. Therefore, we assume that our study provides a sufficiently representative overview of the FI ​​factors existing in the population of CPAS beneficiaries in French-speaking Belgium, except for people over 65 years of age.

For our sampling, all the FI factors existing in the Belgian population were assumed to be found among the beneficiaries of CPAS. However, many people with specific vulnerabilities do not have access to CPAS and could still experience FI related to factors specific to their condition. This could be the case for time limits in workers that earn too much money to benefit from social assistance. It could likewise concern populations that could benefit from social assistance but do not so because of shame or fear, such as undocumented migrants [27]. It is possible that these populations actually experience specific FI factors that were not identified in our study.

Our study was limited to French-speaking Belgium. Considering the few differences in terms of social welfare organization and socio-economic determinants compared to the rest of the country, we believe that our results can easily be generalized to the rest of Belgium.

To our knowledge, our study is the first of its kind to describe the mechanisms underlying FI in Belgium. However, our theoretical framework may need to be completed with the results of other specific populations that were not taken into account in our study.

To our knowledge, our study is the first to have attempted to report, in the most detailed manner possible, the connections between FI factors and food at both the individual and household levels within the Belgian population. This has made it possible to propose a theoretical framework (Fig. 1) for FI at the individual level in our regions.

The concept of “inadequacies” used in our model integrates both individual and framework-dependent factors and suggests that interventions could be made at both levels. This notion was already available in the theoretical framework proposed by the Socialist Mutuality [17], though our model has the advantage of focusing on food security and the points of view of the individual and the household.

Our model confirms the existence, at least in the perceptions of the Belgian population, of several FI factors already confirmed or supposed in the international literature [5, 10–17].

In our model, we categorize food preferences and needs as first determinants of the necessary adaptations and, therefore, of vulnerability to FI. This is in line with the definition of the Rome Declaration, in which emphasis is placed on food preferences and needs [3]. The role of food preferences is illustrated, in particular, by the role of acculturation processes in food security and diet, as revealed in our interviews, thereby corroborating the data retrieved from the literature. Several studies have demonstrated a relationship among migrants between the acculturation level in the destination country, changes in diet, and certain risk factors for chronic diseases and high blood pressure [28].

The lack of difficulties in food availability highlighted in our interviews appears to be consistent with the estimates. In 2011, Eurostat estimated food availability in Belgium at 3793 cal per capita per day [8], well above the 1800–3200 kcal daily amount for an adult recommended by the USDA [29].

In terms of access to food, four factors were highlighted in our interviews: financial, temporal, informational, and a relation to freedom of movement and choice.

Our interviews likely confirmed the existence of financial barriers to access of food. For 12 participants, food and spending that were not constrained and easily adaptable seemed to be used to balance the budget after other constrained expenditures (rent, bills, etc.) or those deemed more important (children, etc.). If the remaining budget was insufficient for a healthy diet, the diet was then adapted, quantitatively or qualitatively, so as not to exceed the budget.

These connections between food prices, lack of financial means, and diet have been described several times in the literature. In 2013, a meta-analysis by Rao et al. showed a median difference of $ 1.5/2000Kcal between the healthiest and least healthy diets [10]. A connection has also been shown between FI for financial reasons and health problems directly related to diet, such as weight gain, diabetes, and cardiovascular risk [19, 30].

For 16 participants, other constraints created an inadequacy by reducing the time available or increasing the time needed to access adequate food according to their needs and preferences. While the role of travel distances and other factors that increase the time required has been cited in the literature [12], no study to date has, to our knowledge, raised the question in terms of the time needed and time available. It seems to us, however, that this way of posing the problem presents the interest on the basis of what is expressed by people in a FI situation. It also brings together many of the mechanisms described, which renders it possible to propose multi-level solutions for different problems.

Six interviews highlighted the role of a lack of information and knowledge regarding the food network as a factor of FI, e.g.*,* during changes in the living environment. Our study is, to our knowledge, the first to describe the role of these determinants. This could have direct implications. For example, proactive information for newcomers to a neighborhood could have beneficial effects on their diet.

Our study proposes the adequation between freedom of choice and movement as well as practical and social constraints as a FI factor. The role of coercion has already been proposed as FI factor in intimate partner violence [15, 16]. Our initial interview matrix and questionnaire did not include questions about coercion, whereas this factor was still mentioned in the interviews. Our interviews have highlighted the potential role of institutional constraints that, to our knowledge, had not yet been explicated in these terms. To pose the problem in these terms, as in our theoretical framework, would make it possible to integrate social constraints like those mentioned in our study (prison, school) and also allow us to integrate material and physical constraints, as in the case of people having lost their capacity to move. Lack of freedom of movement combined with certain social constraints could, for example, be proposed as an explanation for some of the malnutrition among institutionalized geriatric patients [31], who are often limited in their movements and forced to eat the food provided by the institution.

At the level of use, our interviews have highlighted the roles of time, available material, as well as culinary knowledge and skills.

Lack of equipment available to prepare and preserve food, such as observed in homelessness, was described as limiting food options. This notion corroborates the Bocquier et al. results collected in France, which demonstrated an association between lack of cooking equipment and FI [7].

Lack of knowledge and culinary skills likewise appeared to limit food possibilities. The question of lack of culinary skills as a limitation to access to quality food has already been reported in several studies concerning the diet of students and young adults, as found in the review by Munt et al. [13].

The participants described strategies to maintain a food security situation. These included spontaneous strategies related to budget and diet management, as well as the use of informal or institutional aid, all of which had significant limitations.

Three participants emphasized the role of the political and economic framework as a broader determinant of food security. This role is well supported in the literature. In 2016, Loopstra et al. demonstrated a strong correlation in several European countries between a rise of unemployment and decline in wages, on the one hand, and the growth of Eurostat FI indicators, on the other hand. They also showed this correlation to be even lower when per capita social security expenditure proved to be significant [32].

Our questionnaire took into account items that have already been validated in the HFSSM for financial access and in the Radimer/Cornell for physical capacity to shop or cook food [5], though it also added new FI factors identified in other studies [12–17] and in our interviews. Those new items consisted of a general FI screening question, food prices, food availability, physical and geographical access, time, equipment, information, time and ability to use, knowledge, practical and social constraints, and skills. Unlike other questionnaires [5], our new questionnaire was designed to take into account all the FI factors identified in the population in regard to the dimensions of both access and utilization. Our questionnaire had the advantage of being established in the population in which it is intended to be administered, thereby providing a means of obtaining a suitable tool. This new questionnaire still needs further validation in quantitative studies. A more accurate assessment of prevalence would allow thresholds to be set and measurements of different factors to be further increased after using the questionnaire in quantitative studies.

We have already discussed the place that FI could occupy as a determinant of health and how it could interfere with treatments. Therefore, it appears paramount that this dimension be taken into account by the first line of care, at least during any discussion about diet and the management of related pathologies. This could be useful to detect this problem, as is done in other settings and other countries. In adults, the presence of at least one affirmative response to one of the first two questions of the HFSSM (nicknamed the “Hunger Vital Sign”) demonstrated a sensitivity of 97% and a specificity of 92.7% for the FI diagnosis due to financial reasons in the household [33]. In children, these figures were 97 and 87%, respectively, and the presence of at least one affirmative response was associated with a higher risk of poor health of the child as perceived by the parents [34].

The first line of care could also intervene at several levels in managing the FI problem at the individual level. As the first, and at times only, institutional contact of certain vulnerable people in the community, the general practitioner is ideally placed, once the problem is detected, to provide information about the food network and structures and mechanisms for aid and, possibly, to refer them.

Although our work provides a detailed picture of the FI ​​mechanisms that may exist in our country, the data derived from it remain incomplete. Our work was only interested in the point of view of people facing the problem. In an ideal of completeness, these data should be supplemented with other stakeholders and other subjects’ expertise, e.g., focus groups with food aid and health professionals.

Like any qualitative study, our work only highlighted existing subjective experiences, and thereby allowed for the emergence of hypotheses, whereas the elements mentioned here must be confirmed quantitatively. To this end, one possibility could be to test the hypotheses using our questionnaire and other indicators once the theoretical framework is completed.

## Conclusion

We have attempted to summarize the various FI factors that prevail in the Belgian population, as well as the concrete connections that they have with food. These factors were grouped into inadequacies between the resources needed and available in the dimensions of food access (financial, temporal, informational, and freedom factors) and the utilization of food (temporal, material, knowledge, and skills factors).

It appears paramount that this problem be taken into account by the first line of care, not only because it interferes with health status and care, but also because the attending physician is ideally placed to influence the situation. Health professionals, particularly general practitioners, may benefit from identifying this problem in their patients, offering them appropriate counseling and treatment regimens, informing them, and referring them to relevant facilities as appropriate.

In the future, our theoretical model should be further enriched by the experience of the network and quantitatively tested using our questionnaire, inter alia, in correlation with socio-demographic variables and health status. The data obtained would thus offer a clear vision of the FI place in Belgium and, thus, allow for the proposal of solutions adapted to all the potentially involved levels and political, economic, and health systems beyond just simple aid, which is necessary but palliative.

## Supplementary information

**Additional file 1.** Huberland et al. “Questionnaire, initial version”.

**Additional file 2.** Huberland et al. “Questionnaire, final version”.

## Data Availability

The datasets used and analyzed in the course of the study, including transcripts of the interviews, are available from the corresponding author on reasonable request.

## Abbreviations

CPAS: Public Social Assistance Center
DALY: Disability adjusted life years for a given health problem; sum of years of life lost and years of life in poor health impacted by the loss of quality of life
FI: Food insecurity
HFSSM: the Household Food Security Survey Module, a questionnaire used in the United States by the Department of Agriculture for its annual survey on the status of food security in the country; it currently remains the reference indicator
